# Structural Analysis of the 14-3-3ζ/Chibby Interaction Involved in Wnt/β-Catenin Signaling

**DOI:** 10.1371/journal.pone.0123934

**Published:** 2015-04-24

**Authors:** Ryan C. Killoran, Jingsong Fan, Daiwen Yang, Brian H. Shilton, Wing-Yiu Choy

**Affiliations:** 1 Department of Biochemistry, The University of Western Ontario, London, Ontario N6A 5C1, Canada; 2 Department of Biological Sciences, National University of Singapore, 14 Science Drive 4, Singapore, 117543, Singapore; University of Washington, UNITED STATES

## Abstract

The partially disordered Chibby (Cby) is a conserved nuclear protein that antagonizes the Wnt/β-catenin signaling pathway. By competing with the Tcf/Lef family proteins for binding to β-catenin, Cby abrogates the β-catenin-mediated transcription of Wnt signaling genes. Additionally, upon phosphorylation on S20 by the kinase Akt, Cby forms a complex with 14-3-3 to facilitate the nuclear export of β-catenin, which represents another crucial mechanism for the regulation of Wnt signaling. To obtain a mechanistic understanding of the 14-3-3/Cby interaction, we have extensively characterized the complex using X-ray crystallography, nuclear magnetic resonance (NMR) spectroscopy, and isothermal titration calorimetry (ITC). The crystal structure of the human 14-3-3ζ/Cby protein-peptide complex reveals a canonical binding mode; however the residue at the +2 position from the phosphorylated serine is shown to be uniquely oriented relative to other solved structures of 14-3-3 complexes. Our ITC results illustrate that although the phosphorylation of S20 is essential for Cby to recognize 14-3-3, residues flanking the phosphorylation site also contribute to the binding affinity. However, as is commonly observed in other 14-3-3/phosphopeptide crystal structures, residues of Cby flanking the 14-3-3 binding motif lack observable electron density. To obtain a more detailed binding interface, we have completed the backbone NMR resonance assignment of 14-3-3ζ. NMR titration experiments reveal that residues outside of the 14-3-3 conserved binding cleft, namely a flexible loop consisting of residues 203-210, are also involved in binding Cby. By using a combined X-ray and NMR approach, we have dissected the molecular basis of the 14-3-3/Cby interaction.

## Introduction

Chibby (Cby) is a small (14.5 kDa), highly conserved protein that functions as a Wnt/β-catenin signaling antagonist [1]. While the Wnt pathway plays crucial roles in cell proliferation and differentiation, embryonic development and tissue homeostasis, its dysregulation contributes to the pathogenesis of an array of human disorders, including cancer [2,3]. Because of this, pharmacological modulation of the pathway has great therapeutic potential for disease treatments [4,5,6,7].

Activation of the Wnt pathway promotes the stabilization of the transcriptional co-activator β-catenin within the cytoplasm, allowing it to translocate to the nucleus and bind to TCF/LEF (T cell factor/lymphoid enhancer factor) transcription factors to activate Wnt target genes [8,9,10,11]. Multiple regulatory strategies employed by the cell focus on various stages of the Wnt pathway [12], with β-catenin being a focal point. Cby is one such regulatory protein, suppressing β-catenin-mediated signaling via two distinct molecular mechanisms. First, in the nucleus, it competes with the TCF/LEF transcription factors for binding to β-catenin [1]. Secondly, Cby facilitates β-catenin export from the nucleus in conjunction with the proteins 14-3-3 and the nuclear export receptor chromosomal region maintenance 1 (CRM1) [13,14]. In this mode of regulation, Cby forms a stable trimolecular complex with 14-3-3 and β-catenin [13]. Interestingly, binding of 14-3-3 to Cby also leads to an enhanced interaction between Cby and CRM1, promoting nuclear exclusion of Cby-bound β-catenin [14].

The ability of the 126-residue human Cby to function as a scaffold protein likely arises from its partially disordered nature. It is well documented that intrinsically disordered proteins bind to multiple targets by adopting different conformations or via linear motifs located at different regions in the proteins [15,16,17]. Previous work has shown that Cby consists of an unstructured N-terminus and contains a coiled-coil motif within its C-terminus (residues 73–100) that enables dimerization [18,19]. Cby uses its C-terminus to bind to β-catenin [1], although residues critical for this interaction have yet to be elucidated. 14-3-3 recognizes the motif ^16^RKSA(pS)LS^22^ located in the disordered N-terminus of Cby, when the serine 20 residue is phosphorylated by the kinase Akt [13]. In this manner, N- and C-terminal binding modules on Cby work together to facilitate the nuclear export of β-catenin; however, how Cby forms such complexes from a structural standpoint remains unknown. Our work here focuses on elucidating the binding mode between Cby and 14-3-3.

The 14-3-3 proteins, which consist of seven isoforms in mammals (β, ɛ, η, γ, τ, ζ and σ), function as adaptor molecules and are involved in a large range of cellular processes, including apoptosis, DNA damage response and transcriptional regulation [20]. Structurally, the ~30 kDa 14-3-3 proteins assemble into homo- or heterodimers, with each monomer composed of nine anti-parallel alpha-helices (αA-αI) [21]. An amphipathic groove composed of helices αC, αE, αG, and αI comprise the ligand-binding interface. Generally, 14-3-3 isoforms recognize three consensus binding motifs: RXX(pS/pT)XP (mode I), RXXX(pS/pT)XP (mode II) and (pS/pT)X1-2-COOH (mode III) where pS/pT represents phosphorylated serine/threonine [22,23]. However, there are several 14-3-3 binding partners that deviate significantly from these canonical motifs, including examples of non-phosphorylated partners [24,25].

With hundreds of binding partners discovered to date [26], the 14-3-3 proteins are classified as signaling hub proteins. Interestingly, many well-structured hub proteins are found to preferentially bind to unstructured proteins [15]. In fact, the vast majority of 14-3-3 binding motifs are either found or predicted to be within disordered regions [27,28]. While this is due in large part to the phospho-dependence of 14-3-3 interactions, as kinases predominantly target unstructured regions for phosphorylation [29], the structural plasticity of 14-3-3 binding motifs and their neighbouring residues allows 14-3-3 proteins to target many different proteins with high specificity.

14-3-3 targets are hypothesized to bind to 14-3-3 via primary and secondary interactions (discussed by Yang *et al*. [21]). The primary interactions are made between the 14-3-3 binding motif (including the pS/pT residue) of the target and a conserved binding groove in the 14-3-3 protein. Secondary interactions occur when residues flanking the target’s 14-3-3 binding-motif contact regions outside of the conserved 14-3-3 binding groove. While various crystal structures of 14-3-3 complexes have been solved, the majority of them have been co-crystallized with short peptides. Observable electron density is typically only present for 4–10 residues flanking the phosphorylated residue of the target peptide. While this provides a structural basis for the primary contacts of the interaction, information regarding the secondary contacts is limited. To date, only one (nearly) full-length, globular partner has been co-crystallized with 14-3-3 [30].

The disordered Cby is able to interact with at least three of the seven 14-3-3 isoforms (ɛ, η and ζ) [13]. Its 14-3-3 binding motif (^16^RKSA(pS)LS^22^) closely resembles the mode II motif (RXXX(pS/pT)XP), the difference being that Cby contains a serine instead of proline at the +2 position from the phosphoserine. It is noteworthy that even though proline is found to be the +2 residue in ~ 50% of known 14-3-3 binding motifs, serine is in fact the second most commonly found amino acid at that position [25]. To our knowledge, no 14-3-3/phosphopeptide structure with serine as the +2 residue has been determined to date.

In this work, the crystal structure of 14-3-3ζ in complex with an 18-residue phosphorylated Cby-derived peptide was solved to reveal the primary binding interactions. Secondary interactions between 14-3-3 and the Cby peptide were further investigated by using nuclear magnetic resonance (NMR) spectroscopy and isothermal titration calorimetry (ITC). By combining the X-ray and NMR techniques, we were able to obtain additional molecular details of the binding interface of Cby on 14-3-3. Notably, the backbone assignment of 14-3-3ζ completed in this work will also facilitate mapping of regions on the 14-3-3ζ surface that are involved in binding other targets.

## Results

### Phosphorylation of serine 20 on Cby is critical for its binding to 14-3-3ζ

A previous study by Li *et al*. [13] showed that S20 of Cby is essential for its interaction with 14-3-3, with the binding being regulated by the phosphorylation of this serine residue. To quantitatively assess the importance of the phosphorylation of S20 to the complex formation with 14-3-3ζ, we have used ITC to measure the affinities of a Cby peptide (^12^KTPPRKSASLSNL^24^) encompassing the 14-3-3 binding motif, in both its non-phosphorylated and phosphorylated forms, to 14-3-3ζ. The same peptides containing the phospho-mimetic mutants S20D and S20E were also included in this study. Our data show that only the phosphorylated Cby peptide was able to interact with 14-3-3ζ (K_D_ ~ 15 μM, Table 1), while the other three peptides display no observable binding (S1 Fig). Importantly, the phospho-mimetic mutations were unable to rescue the binding, indicating that the Cby/14-3-3ζ association is driven by the phosphate group on S20.

**Table 1:** Thermodynamic parameters for the binding of phosphorylated Cby peptides to 14-3-3.

14-3-3 construct in ITC Cell	Peptide	n^a^	K_d_ ^b^ (10^-6^ M)	ΔH^b^ (kcal/mol)	TΔS^b^ (kcal/mol)	ΔG^b^ (kcal/mol)
14-3-3ζ						
	Cby 7-mer^16^RKSA(pS)LS^22^	1.01	43.5 ± 1.9	-3.35 ± 0.06	2.60	-5.95 ± 0.03
	Cby 11-mer^12^KTPPRKSA(pS)LS^22^	0.95	17.5 ± 0.6	-3.26 ± 0.02	3.23	-6.49 ± 0.02
	Cby 13-mer^12^KTPPRKSA(pS)LSNL^24^	1.00	14.5 ± 0.6	-2.63 ± 0.02	3.97	-6.60 ± 0.02
	Cby 18-mer^12^KTPPRKSA(pS)LSNLHSLDR^29^	0.96	4.6 ± 0.2	-4.35 ± 0.05	2.93	-7.28 ± 0.03
	Cby 7-mer S22P^16^RKSA(pS)LP^22^	1.03	2.9 ± 0.05	-6.16 ± 0.01	1.39	-7.55 ± 0.01
	Cby 13-mer S22P^12^KTPPRKSA(pS)LPNL^24^	1.06	1.1 ± 0.03	-4.64 ± 0.01	3.49	-8.13 ± 0.02
	Cby 18-mer S22P^12^KTPPRKSA(pS)LPNLHSLDR^29^	1.05	0.36 ± 0.01	-8.03 ± 0.02	0.76	-8.79 ± 0.02
	Cby 18-mer L24A^12^KTPPRKSA(pS)LSNAHSLDR^29^	0.99	21.3 ± 1.7	-1.56 ± 0.05	4.81	-6.37 ± 0.05
14-3-3ζ K49A						
	Cby 18-mer WT^12^KTPPRKSA(pS)LSNLHSLDR^29^	1.01	3.8 ± 0.3	-4.16 ± 0.05	3.23	-7.39 ± 0.05
	Cby 18-mer S22P^12^KTPPRKSA(pS)LPNLHSLDR^29^	1.07	1.9 ± 0.2	-7.13 ± 0.03	0.67	-7.80 ± 0.06
14-3-3ζΔC12						
	Cby 13-mer^12^KTPPRKSA(pS)LSNL^24^	0.99	14.9 ± 0.9	-2.08 ± 0.03	4.50	-6.58 ± 0.04
	Cby 18-mer^12^KTPPRKSA(pS)LSNLHSLDR^29^	1.04	6.8 ± 0.4	-3.35 ± 0.06	3.70	-7.05 ± 0.03

A duplicate set of experimental values for all ITC experiments is reported in S1 Table.

^a^ Binding stoichiometry of monomeric 14-3-3 and Cby peptide.

^b^K_d_ is the dissociation constant ΔH, ΔS and ΔG are the change in enthalpy, entropy and Gibbs free energy upon binding at T = 298.15 K, respectively.

### Flanking residues of the consensus 14-3-3 binding motif play important roles in the Cby/14-3-3ζ association

Next, we assessed the contributions of residues flanking the consensus 14-3-3 binding motif of Cby to the interaction with 14-3-3ζ. The results of our ITC measurements show that a 7-mer Cby peptide (^16^RKSA(pS)LS^22^) comprising the minimal 14-3-3 binding motif bound to 14-3-3ζ with a K_D_ of ~44 μM (Table 1, S2 Fig). Interestingly, phosphopeptides of increasing length exhibit a systematic increase in binding affinity to 14-3-3ζ (the results are summarized in Table 1 and the thermograms are shown in S2 Fig). First, a Cby 11-mer peptide which contains 4 additional residues (^12^KTPP^15^) than the Cby 7-mer peptide (^16^RKSA(pS)LS^22^) bound with more than two-fold greater affinity than the Cby 7-mer, with a K_D_ of ~18 μM. A Cby 13-mer, with a 2 residue C-terminal extension to the 11-mer (^23^NL^24^), bound with only slightly higher affinity, with a K_D_ of ~15 μM. Lastly, an 18-mer, which contains a five-residue C-terminal extension to the 13-mer (^25^HSLDR^29^), bound with nine-fold greater affinity compared to the 7-mer, with a K_D_ of ~5 μM. The results clearly indicate that residues flanking the binding-motif on Cby play significant roles in mediating the interaction with 14-3-3ζ.

### Molecular basis of the interaction between 14-3-3ζ and the non-canonical mode II binding-motif of Cby

**Fig 1:**
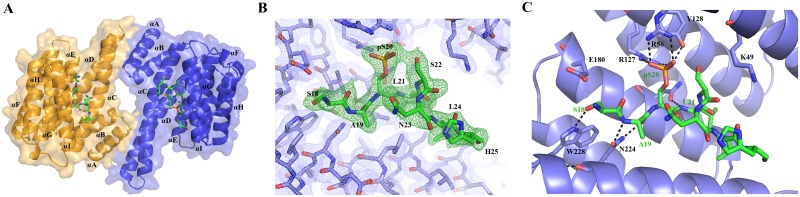
(A) Crystal structure of the 14-3-3ζ /Cby 18-mer complex. Each 14-3-3ζ monomer (coloured orange and blue) is bound to one Cby phosphopeptide. (B) Final 2*F*_o_ − *F*_c_ electron density of 14-3-3ζ (blue mesh) and Cby 18-mer (green mesh) contoured to 1σ. (C) Polar contacts (black hashed lines) between 14-3-3ζ residues (light blue sticks) and the Cby 18-mer (green sticks).

We used X-ray crystallography to elucidate the molecular basis of the 14-3-3ζ/Cby interaction. Purified 14-3-3ζ protein was co-crystallized with the phosphorylated Cby 18-mer (^12^KTPPRKSA(pS)LSNLHSLDR^29^) and diffraction data were collected to a resolution of 2.41 Å (Table 2). The 14-3-3ζ protein, which crystallized as the typical cup-shaped dimer [20], has the Cby peptide bound in an extended conformation to each 14-3-3 monomer (Fig 1A). The dimer comprises the asymmetric unit of the crystal, and the visible electron density was slightly more extensive for the peptide bound to the B-chain of the dimer. Thus, of the 18 residues in the Cby peptide, eight residues, ^18^SA(pS)LSNLH^25^, were resolved in the peptide bound to the 14-3-3 B-chain (Fig 1B) and seven residues, ^18^SA(pS)LSNL^24^, for that bound to the 14-3-3 A-chain (not shown). Both bound peptides adopted similar conformations and made the same interactions with the 14-3-3 protein. The lack of observable electron density for the remaining residues suggests that they remain flexible within the complex.

**Table 2:** Crystallographic data collection and refinement statistics.

*Data Collection*	
Wavelength (Å)	1.54179
Cell Parameters	
*a*, *b*, *c* (Å)	70.83, 71.96, 130.99
Space group	P2_1_2_1_2_1_
Resolution (Å)	65.49–2.41 (2.53–2.41)
Total reflections	139,670 (16,075)
Unique reflections	26,474 (3445)
R_merge_	0.072 (0.516)
Completeness (%)	98.4 (89.5)
Multiplicity	5.3 (4.7)
I/σ(I)	13.9 (3.2)
*Refinement*	
R_work_/R_free_ (%)	0.203/0.256
RMSD from ideal values	
Bonds (Å)	0.008
Angles (°)	1.12
Overall mean B values (Å^2^)	
Protein	21.71
Peptide	26.69
Solvent	19.22
Number of amino acid residues per asymmetric unit	464
Number of water molecules	147
Ramachandran plot	
Favoured regions (%)	98
Allowed regions (%)	1.8
Disallowed regions (%)	0.2
Cβ deviations greater than 0.25 Å	0

Values in parentheses refer to the highest resolution shell.

The overall structural features of the 14-3-3ζ/Cby complex are similar to that of other 14-3-3ζ/phosphopeptide complexes. The Cby peptide occupies the conserved binding groove of 14-3-3 with the same orientation as other phosphopeptides previously characterized. The hydrogen bonding potential of the phosphate group on pS20 is fully realized, with two hydrogen bonds made to each of R56 and R127, and another hydrogen bond to the side-chain hydroxyl of Y128 (Fig 1C); 3 water molecules and the side chain of Cby-S22 mediate four additional hydrogen bonds to the phosphate oxygens. Thus, each of the phosphate oxygens has 3 hydrogen bonding partners positioned in an ideal tetrahedral arrangement, explaining why mutation of S20 to D or E fails to promote high affinity binding of Cby to 14-3-3ζ. The peptide is further coordinated by hydrogen bonds between the side-chain of 14-3-3ζ-N224 with the backbone nitrogen and carbonyl of Cby-A19, the carboxamide of 14-3-3ζ-N173 to the backbone nitrogen of Cby-L21, and the side-chain of either E180 (chain-C) or W228 (chain-D) of 14-3-3ζ to the hydroxyl group on the side-chain of Cby-S18. Intra-chain contacts exist within the Cby peptide as well. On chain-D of the model, consisting of residues ^18^SA(pS)LSNLH^25^, the pS20 amide is hydrogen bonded to the N23 side-chain, the phosphate group itself on S20 hydrogen bonds with the hydroxyl group on the S22 side-chain, the pS20 carboxyl contacts both the amide and the side-chain of N23 and the carboxyl of L21 contacts the amide of L24.

### An S22P mutation to Cby enhances its interaction with 14-3-3ζ

**Fig 2:**
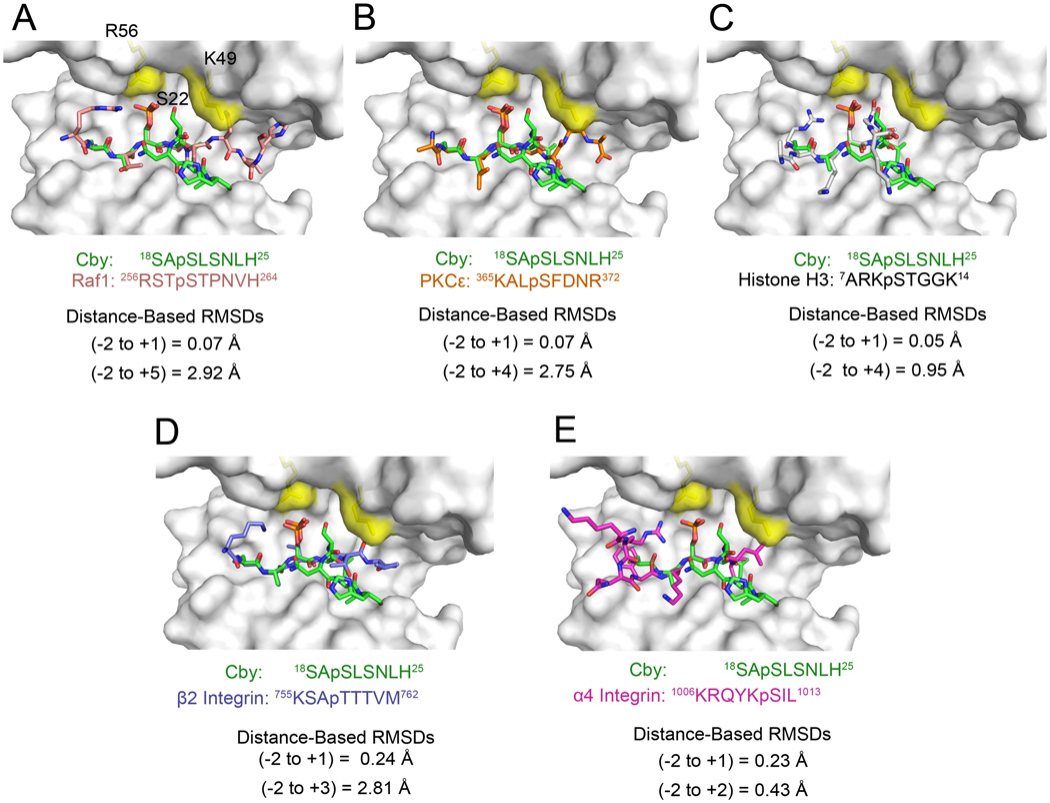
Structural comparison of 14-3-3ζ-bound Cby with other 14-3-3 binding motifs comprising various +2 residues. Residues K49 and R56 are coloured yellow on the white surface representation of 14-3-3ζ. Structural and sequence alignment of Cby (green sticks) with the binding-motifs of (A). Raf1 (pink sticks, PDB: 3CU8), (B) PKCε (orange sticks, PDB: 2WH0), (C) Histone H3 (white sticks, PDB: 2C1N), (D) β2 integrin (blue sticks, PDB: 2V7D) and (E) α 4 integrin (purple sticks. PDB: 4HKC). The Cα RMSD values were computed by subtracting a Cα distance matrix between the -2 and +1 residues of Cby and each peptide as well as the -2 and most C-terminal residue in the respective alignments.

As mentioned, Cby comprises a serine (S22) in the +2 position to the phosphoserine, as opposed to the canonical proline. A comparison between the 14-3-3ζ/Cby structure with other 14-3-3ζ/phosphopeptide complexes with different +2 residues [including the canonical proline (Raf1), aspartate (PKCε [31]), glycine (Histone H3 [32]), threonine (β2 integrin [33]) and leucine (α4 integrin [34])], reveals that the S22 side chain is oriented uniquely, projecting between 14-3-3ζ residues R56 and K49 (Fig 2). Also, superimposition of these peptides onto Cby demonstrates that the backbones of residues -2 to +1 typically align well but diverge quickly C-terminally from the +2 residue.

With the side chain on Cby-S22 distinctively positioned relative to other +2 residues in 14-3-3/phosphopeptide structures, we sought to determine if mutating this serine to the canonical proline would have a significant effect on Cby’s interaction with 14-3-3ζ. The same 7-mer, 13-mer and 18-mer Cby phosphopeptides used previously, but now containing the S22P mutation, were used in ITC experiments to determine the binding affinity to 14-3-3ζ (Table 1, S2 Fig). Interestingly, for all S22P Cby peptides, the binding affinity was approximately 15-fold tighter for each peptide respective to its wild-type counterpart (for 7-mer, K_D_ ~3 versus ~44 μM; for 13-mer, K_D_ ~1 versus ~15 μM; for 18-mer, K_D_ ~360 nM versus ~5 μM). The increases in binding affinity for the three S22P mutant peptides were all due to more negative ΔH of binding. Moreover, the flanking residues within the S22P Cby peptides, like the WT, appear to play a role in the interaction as well. The Cby S22P 18-mer bound ~9 fold tighter than the S22P 7-mer, similar to the difference observed between the WT Cby 18-mer and 7-mer peptides.

In numerous crystal structures of 14-3-3 proteins in complex with phosphorylated S/T peptides, the conserved K49 (numbering refers to the ζ isoform) side-chain is observed forming a hydrogen bond with the phosphate group on pS/pT (illustrated in S3 Fig with an elucidated 14-3-3ζ/Raf1 structure). With Cby’s S22 side-chain juxtaposed between 14-3-3ζ residues K49 and R56, the K49 side-chain is sterically hindered from contacting pS20. As such, we speculated that the increases in binding affinity to 14-3-3ζ observed for the S22P mutant peptides might be the result of the favorable contacts between K49 and pS20 facilitated by the proline-induced conformational changes. To test this, a K49A mutation was introduced into 14-3-3ζ and ITC titrations were performed with 18-mer WT and S22P Cby peptides. Interestingly, the data show that the K49A mutation only had a minor effect (ΔΔG ~ 0.1 kcal/mol) on the binding to the WT Cby peptide, however, the mutant bound with lower affinity to the S22P Cby peptide compared to the WT 14-3-3ζ (ΔΔG ~ 1.0 kcal/mol) (Table 1, S4 Fig).

### Probe for secondary interactions between 14-3-3ζ and Cby using NMR spectroscopy

The crystal structure of 14-3-3ζ/Cby complex clearly reveals the molecular basis of primary interactions between 14-3-3ζ and the non-canonical mode II binding motif within Cby. However, like most of the 14-3-3/phosphopeptide structures available to date, the lack of electron density for residues outside the phosphorylated binding motif of Cby hinders our understanding of the secondary interactions in complex formation. Notably, our ITC results showed that the Cby 18-mer (^12^KTPPRKSA(pS)LSNLHSLDR^29^) used in the crystallographic study has a significantly higher affinity compared to the 7-mer (^16^RKSA(pS)LS^22^; the minimal 14-3-3 binding motif) for 14-3-3ζ. However, only residues ^23^NLH^25^ outside the minimal binding motif were observable in the crystal structure. Of the three, only the L24 side-chain is buried within the interface, projecting toward a hydrophobic patch consisting of 14-3-3ζ residues L216 and I217. Intriguingly, immunoprecipitation experiments performed by Li *et al*.[14] demonstrated that an alanine substitution at L24 on Cby abolished binding to 14-3-3ζ. Our ITC titrations with the Cby 18-mer comprising the L24A mutation revealed that its interaction with 14-3-3ζ was indeed impaired but not abolished, binding approximately 4-fold weaker than the wild-type peptide with a K_D_ ~21 μM (Table 1, S2 Fig). To gain a more thorough understanding of the secondary interactions between 14-3-3ζ and Cby, we sought to determine if there are additional regions in 14-3-3ζ that interact with the Cby 18-mer compared to the 7-mer using NMR chemical shift mapping, a powerful technique for identifying binding interfaces in solution.

Due to the relatively high molecular weight of the 14-3-3ζ dimer (~56 kDa), deuterated protein samples were used in our NMR studies. An initial ^1^H-^15^N TROSY-HSQC spectrum of 14-3-3ζ displayed many well-dispersed resonance signals along with a small number of extremely intense peaks (S5 Fig). These strong peaks do not display any chemical shift changes upon titration of the unlabeled 18-mer Cby peptide, indicating that their corresponding residues are not involved in the interaction (S5 Fig). However, their presence in the ^1^H-^15^N TROSY-HSQC spectrum unavoidably complicates the spectral analysis by masking many underlying resonances. We speculated that these high intensity resonances originate from 14-3-3ζ’s C-terminal tail, which has been shown to be disordered [35]. With this in mind, we have generated the 14-3-3ζΔC12 construct, which is full-length 14-3-3ζ with the C-terminal 12 residues being removed. The ^1^H-^15^N TROSY-HSQC of 14-3-3ζΔC12 overlays almost perfectly with that of 14-3-3ζ, except that the highly intense peaks are no longer observable, confirming that these resonances originate from the C-terminal tail. Importantly, ITC experiments show that 14-3-3ζΔC12 binds to Cby peptides with almost the same affinity as full-length 14-3-3ζ (Table 1, S6 Fig).

**Fig 3:**
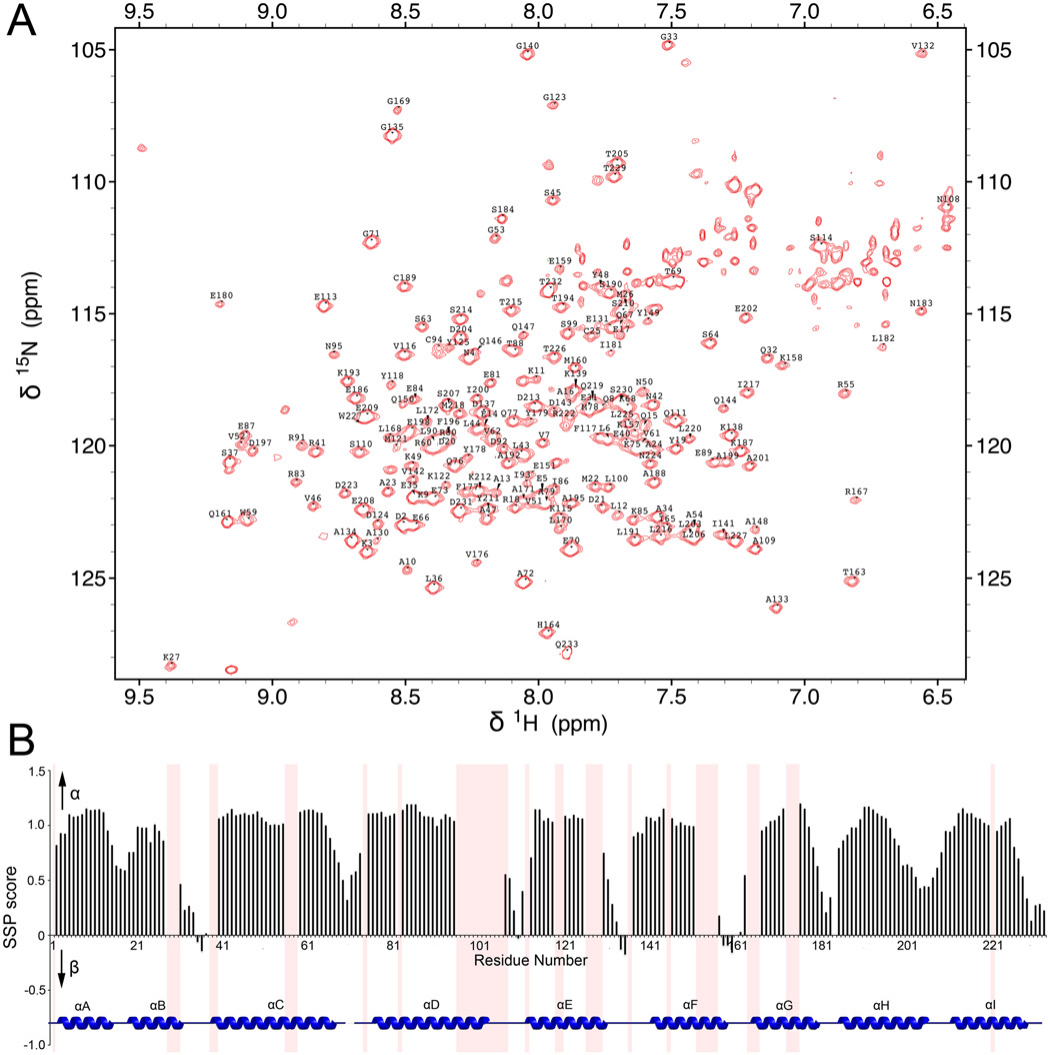
(A) ^1^H-^15^N TROSY-HSQC spectrum and backbone resonance assignment of ^2^H/^13^C/^15^N labeled 14-3-3ζΔC12. (B) Secondary structure propensity (SSP) scores for 14-3-3ζΔC12. SSP scores were calculated based upon the ^13^Cα/β chemical shifts.

Backbone resonance assignments for ^1^H^N^, ^15^N, ^13^Cα and ^13^Cβ were obtained for 14-3-3ζΔC12 (Fig 3A). We were able to assign 82% of the ^1^H^N^ and ^15^N resonances of non-proline residues, 81% of all ^13^Cα and 81% all ^13^Cβ resonances. The presence of weak peaks prevented a higher percentage of resonance assignments. The residue-specific secondary structure propensity (SSP) scores using ^13^Cα/β chemical shifts [36] indicate that the protein is, as expected, largely α-helical (Fig 3B).

### Mapping the Cby peptides binding regions on 14-3-3ζΔC12

**Fig 4:**
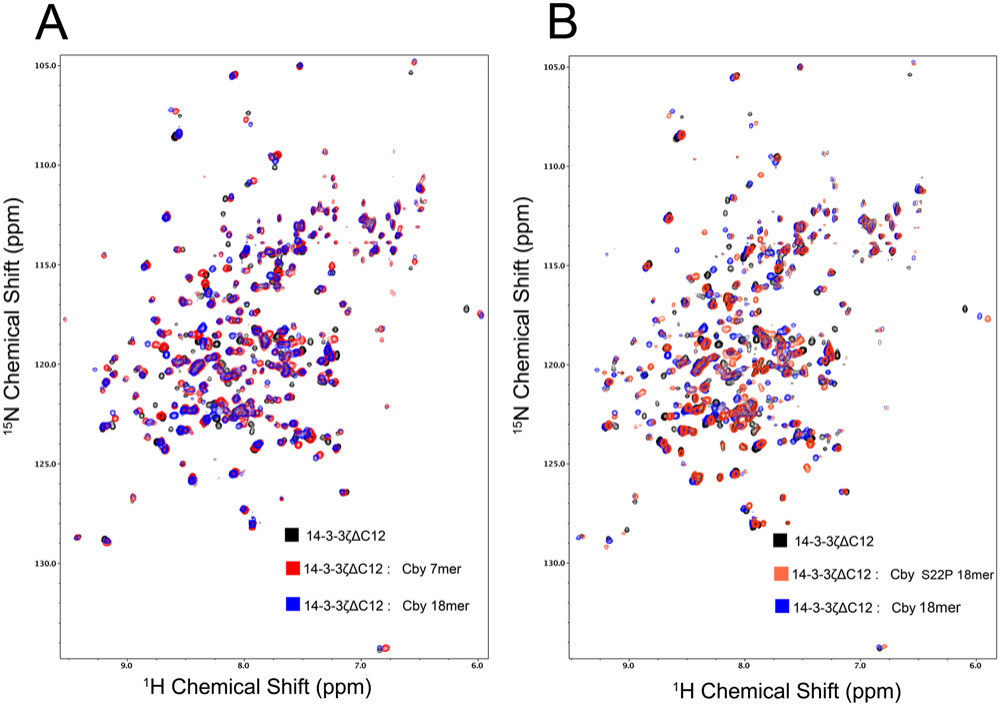
NMR titration experiments of 14-3-3ζΔC12 with Cby peptides. (A) ^1^H-^15^N TROSY-HSQC spectra of 14-3-3ζΔC12 alone (black) and with 3 molar equivalents of the Cby 7-mer (red) and Cby 18-mer (blue). (B) ^1^H-^15^N TROSY-HSQC spectra of 14-3-3ζΔC12 alone (black) and with 3 molar equivalents of the WT Cby 18-mer (blue) and Cby S22P 18-mer (blue).

**Fig 5:**
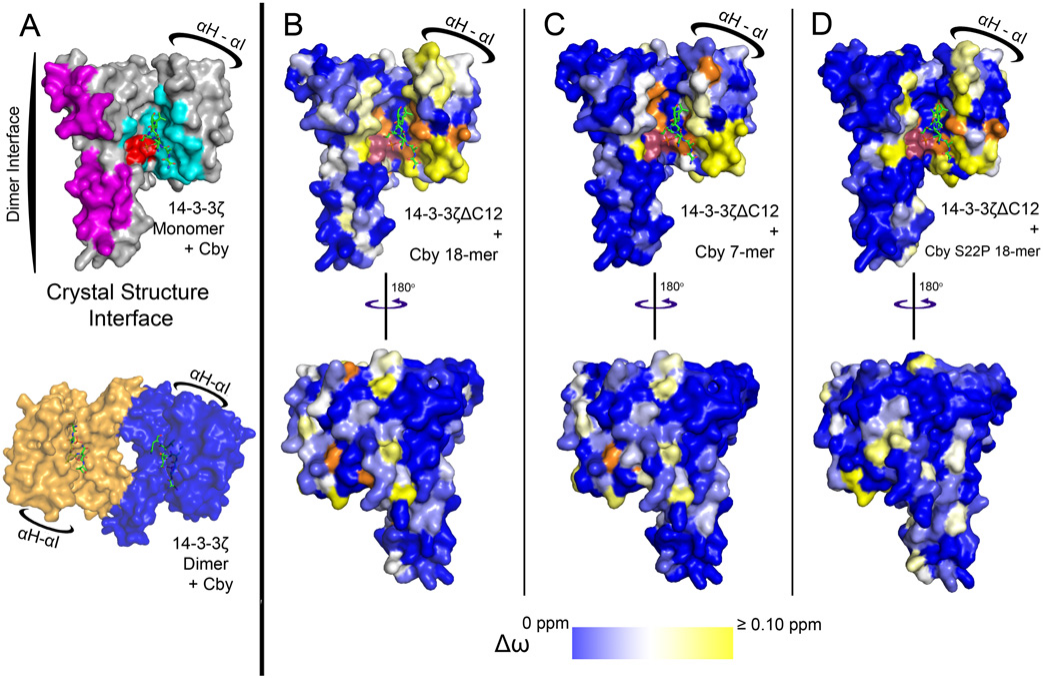
The mapping of chemical shifts on the crystal structure of the 14-3-3ζ /Cby complex. Due to the crowding of some peaks, the chemical shifts of some residues could not be confidently traced and were excluded from the analysis. (A) (Above) A monomer of the 14-3-3ζ /Cby crystal structure interface based on Cby residues (^18^SApSLSNLH^25^). Residues R56, R127 and Y128 which contact the Cby phosphate group are coloured red, residues interfacing the peptide are coloured cyan, and 14-3-3ζ residues coloured magenta are found along the 14-3-3 dimer interface. (Below) The 14-3-3ζ dimer bound to Cby. (B) (Cby 7-mer), (C) (Cby 18-mer) and (D) (Cby S22P 18-mer). Residues with traceable assigned resonances are coloured on a blue—white—yellow gradient (0 ppm to 0.1 ppm) based on their combined chemical shift [Δω = ((Δδ^1^H)^2^ +(0.2*Δδ^15^N)^2^)^1/2^] at a 3:1 peptide:protein ratio. Residues coloured in orange represent peaks that broadened out to disappearance upon addition of peptide. Residues R56, R127 and Y128 are coloured pink.

The nearly complete backbone assignment of 14-3-3ζΔC12 affords an opportunity to map the binding regions of different Cby peptides on 14-3-3ζ in a highly efficient manner. The 7-mer and 18-mer Cby peptides were titrated to 14-3-3ζΔC12 in a series of NMR experiments to a final molar ratio of 3:1 (peptide:protein) for chemical shift mapping (Fig 4A). When comparing the ^1^H-^15^N TROSY-HSQC spectra of 14-3-3ζΔC12 in the apo and the 7-mer/18-mer Cby bound forms, a large number of chemical shift changes as well as peak broadenings are observed. The chemical shift perturbations were calculated for both titration sets and were mapped onto the 14-3-3ζ/Cby structure (Fig 5B & 5C, S7A Fig). From the generated maps, the residues exhibiting the largest chemical shift changes for both peptides are found within helices αC, αE and αI, matching well with the binding interface observed in the crystal structure (Fig 5A). Additional peaks within the binding cleft were broadened out over the course of the titrations. For the Cby 18-mer titration, these peaks include M121, D124, Y125 and A130 within αE, L170, L172, V176 and Y178 within αG, and I217, L220 and R222 within αI. Broadened resonances within the binding cleft are similar for the Cby 7-mer titration, including F117, M121, D124 and A130 within αE, L170, L172, V176, F177 and Y178 within αG, and Y211 within αI. Even though residues that bond with the phosphate group of S20 on Cby, R56, R127 and Y128, could not be assigned, residues surrounding this basic patch either broaden out (M121, D124, Y125, V176) or exhibit large chemical shift changes (V52, G53) as expected. Large chemical shift perturbations or broadened resonances were not observed along the 14-3-3ζ dimer interface for either peptide.

Notably, the Cby 18-mer binding interface appears to be larger than that of the pCby 7-mer. Many 14-3-3ζ residues within the loop between αH and αI (204–208), along with N-terminal residues of αI (209–215) exhibit larger chemical shift perturbations when titrated with the Cby 18-mer compared to the Cby 7-mer (S8 Fig). Based on the orientation of the peptide, we speculate that this region of 14-3-3 interacts with the C-terminal (^26^SLDR^29^) residues on the Cby 18-mer peptide.

Additional NMR titration experiments were performed with the Cby S22P 18-mer to compare its binding interface with the WT. The chemical shift analysis (S7B Fig) and subsequent mapping onto the 14-3-3ζ/Cby structure (Fig 5D) revealed a similar interface to that observed for the Cby WT 18-mer. Interestingly, while the resonance signals of some residues in the αH—αI loop and residues within the 209–215 range in αI shift similarly when titrated with the WT or S22P peptides, the T205, L206, E209 and S210 peaks have much smaller shifts when titrated with the Cby S22P 18-mer. Having a proline at the +2 position may affect how the downstream C-terminal residues interact with this loop region.

## Discussion

The molecular basis of the interaction between 14-3-3ζ and Cby was extensively investigated by using a combination of X-ray crystallography, NMR spectroscopy, and ITC. Our ITC results show that even though phosphorylation of Cby-S20 is critical for the complex formation (as Cby peptides comprising S20D and S20E phospho-mimetics failed to bind to 14-3-3ζ), residues outside the 14-3-3 binding-motif also play an important role in the interaction. This is supported by the finding that a short, 7-residue phosphopeptide of Cby comprising the minimal 14-3-3 binding-motif bound 10-fold weaker compared to a longer, 18-residue phosphopeptide. A similar observation was made in a recent study of 14-3-3/α-integrin tail complexes, demonstrating that a 30-residue α4-integrin peptide comprising its 14-3-3-binding motif bound up to 15 times tighter to 14-3-3ζ than an 11-residue peptide [34]. Interestingly, in the same study, short and long phosphopeptides comprising the 14-3-3 binding motif for the β2-integrin bound with nearly the same high affinity, suggesting that secondary interactions from flanking residues are likely sequence specific.

Another interesting finding from our ITC experiments is that the phosphorylated Cby peptides comprising a mutation at the pS + 2 position to the canonical proline (S22P) bind ~15 fold tighter than the WT peptides. Our crystal structure provides molecular insight into the target binding specificity of 14-3-3. Based on available crystal structures of various 14-3-3 isoforms in complex with phosphorylated peptides comprising type I or type II binding motifs (for example, PDB: 1QJA [37], 1QJB [37], 3MHR [38]), the K49 (ζ sequence) side chain commonly forms a hydrogen bond with the phosphate group on pSer. However, in the 14-3-3ζ/Cby crystal structure, the side chain of S22 positions between K49 and R56 of 14-3-3ζ and sterically hinders the interaction between K49 and pS20. Interestingly, introducing K49A mutation to 14-3-3ζ does not affect its affinity for the WT Cby peptide, but the binding affinity to the S22P peptide was reduced by ~5 fold. From these results, we speculate the S22P mutation of Cby promotes a conformational change in the bound state, allowing K49 on 14-3-3ζ to form hydrogen bond with the phosphate group on S20. However, it should be noted that there are examples of peptides containing a +2 proline co-crystallized with 14-3-3 proteins where K49 is either not making any direct contacts with the ligand (PDB: 4FL5) or it interacts with other residues within the phosphopeptides (PDB: 3UAL [39], 4IEA [40]) instead of the phosphorylation site. Additionally, a proline at the +2 position is not absolutely essential for a K49—phosphate group interaction. This contact is observed in the 14-3-3γ/tyrosine hydroxylase structure (PDB: 4J6S [41]) and in the 14-3-3ζ/alpha-4 integrin, which both have a leucine at the +2 position.

With only 8 of the 18 residues in the Cby 18-mer peptide resolved in the crystal structure, it is likely that any secondary contacts made by flanking residues are very dynamic. This is a common issue in the co-crystallization of 14-3-3 proteins and peptides. Typically, only short regions (4–10 residues comprising residues nearby the pS/pT) within longer phosphopeptides are resolved. Because significant secondary interactions may be critical to 14-3-3ζ’s interaction with Cby, we elected to pursue NMR spectroscopy as a means of further characterizing the complex.

Chemical shift analysis of ^1^H-^15^N TROSY-HSQC titration experiments, performed by titrating the 7-mer and 18-mer Cby peptides into isotopically labeled 14-3-3ζΔC12, allowed for chemical shift mapping to reveal any differences in the binding interface between both peptides. We found that 14-3-3ζ residues 204–215 are involved in the interaction with the Cby 18-mer but not the 7-mer. Based on the orientation of the peptide in the crystal structure, this would correspond to the residues C-terminal to the pS20, ^23^NLHSLDR^29^, making transient contacts with this region of 14-3-3ζ. Chemical shift analysis of the Cby S22P 18-mer bound to 14-3-3ζΔC12 revealed a similar binding site to that of the WT 18-mer, however some variability in shifts for residues within the 204–215 range were observed. This suggests that the +2 residue can affect how downstream C-terminal residues interact with 14-3-3ζ. A +2 proline is of particular interest because while it is most commonly observed bound in a *trans* conformation (e.g. 1QJA [37]) it has been observed bound in the *cis* conformation (e.g. 1QJB [37]).

Residues 203–210, corresponding to 14-3-3ζ’s αH- αI linker, have been shown to be part of a secondary binding site with another 14-3-3 target [30]. The crystal structure of the 14-3-3ζ/phosphorylated-AANAT complex, the only full-length protein to be solved in complex with 14-3-3, demonstrates that this linker is used by 14-3-3ζ to bind AANAT, highlighted by the 14-3-3ζ residue E208 making a salt bridge with R53 of phosphorylated AANAT, located in the globular region of the protein. Additionally, this linker region has been implicated in ligand discrimination between 14-3-3 isoforms [42,43]. For instance, the phosphatase Cdc25C can bind to all 14-3-3 isoforms except for σ; however, the mutation of divergent residues within 14-3-3σ’s αH- αI linker to those conserved across all other isoforms enables the engineered σ to bind Cdc25C [43].

**Fig 6:**
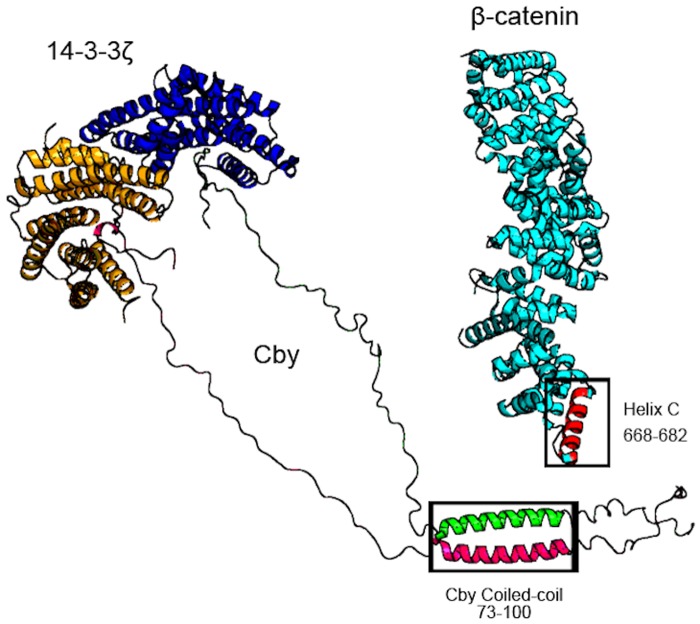
The 14-3-3ζ, Cby, β-catenin tripartite complex. Model of dimeric 14-3-3ζ bound to two molecules of full-length Cby. The models of two full-length Cby proteins have disordered residues 30–73 of the N-terminus and 100–126 of the C-terminus presented in a highly extended fashion, and are shown forming a coiled-coil from residues 73–100. β-catenin (PDB: 2Z6H) is shown with its Cby binding-site (helix C) in red which then binds along the C-terminus (64–126) of Cby.

Building on this work, future studies are in progress to elucidate how the complex forms in the context of full-length Cby. It is conceivable that two Cby molecules could retain a coiled-coil structure while each is bound to 14-3-3ζ (Fig 6). Because of the orientation each 14-3-3ζ monomer adopts upon dimerization, the Cby residues lying on the C-terminal end of serine 20 will exit the binding interface at opposing ends of the 14-3-3ζ dimer. Since the N-terminal residues (1–64) of full-length Cby are disordered [19] it is feasible that each bound Cby molecule retains the flexibility necessary to orient itself to form a coiled-coil from residues 73–100, as well as to bind to the 88-kDa β-catenin. The model (Fig 6) is consistent with the results of Li *et al*.[13], which demonstrated that binding of Cby to β-catenin is not interfered by the increased level of 14-3-3. We are aware that a Cby dimer binding to the 14-3-3 dimer could presumably lead to a stronger interaction between the two molecules than what was reported in this study due to avidity. How the complex ultimately forms, i.e. its stoichiometry and conformation, will aid in providing the mechanistic basis by which the two proteins recruit β-catenin to form a trimolecular complex and promote β-catenin’s nuclear exclusion.

Also of interest is the interplay of Cby’s interactions with 14-3-3 and CRM1. Cby’s 14-3-3 binding motif is juxtaposed with its nuclear export signal (NES) (^21^LSNLHSLDR^29^). Immunoprecipitation experiments by Li *et al*. [14] revealed that 14-3-3 enhanced the Cby-CRM1 interaction in a dose-dependent manner. It was postulated that 14-3-3 might induce a conformational change in Cby to expose its NES for CRM1 recruitment. In doing so, CRM1 could then mediate the nuclear exclusion of Cby. Our crystal structure demonstrates that this mechanism may not be the case. The consensus NES sequence is Φ^1^(X_2–3_)Φ^2^(X_2–3_)Φ^3^XΦ^4^, where Φ represents critical hydrophobic residues L, I, V, F or M and X is any amino acid[44,45]. In determined crystal structures of CRM1/target complexes, the critical hydrophobic residues are buried in a hydrophobic cleft formed by HEAT repeats 11 and 12 of CRM1[46,47]. The hydrophobic residues on Cby’s NES correspond to L21, L24 and L27. From our 14-3-3/Cby crystal structure, the L21 and L24 sidechains are projected toward 14-3-3’s binding cleft, making them inaccessible for CRM1. Residues (^26^SLDR^29^) are unobservable in our structure and thus their accessibility remains unknown. However, Li *et al*. [14] demonstrated that a L27A mutation to Cby did not affect the Cby/CRM1 interaction, and showed normal, WT subcellular distribution. Meanwhile, L21A and L24A mutations abolished the interaction with CRM1 and led to the nuclear sequestration of Cby. As such, 14-3-3 may need to be dissociated from Cby to allow for the CRM1 interaction. Further cell experiments and structural studies of the Cby/CRM1 complex are needed to comprehend how 14-3-3 and CRM1 work in concert to promote the cytoplasmic sequestration of Cby.

Finally, NMR studies are critical to the characterization of 14-3-3 interactions as their interactome is predicted to predominantly consist of disordered targets [27]. The backbone resonance assignment of 14-3-3ζ completed in this work therefore not just facilitate the studies of 14-3-3ζ with Cby, it will be invaluable for probing interactions between 14-3-3ζ and a continually growing list of protein targets by NMR. Such studies may allow the identification of ligand-binding interfaces on 14-3-3 in an efficient manner as illustrated in this work. Moreover, with 14-3-3ζ over-expression associated with tumour progression and chemoresistance (reviewed in Matta *et al*. [48]), the protein is an attractive therapeutic target in cancer. With the availability of the backbone resonance assignment of 14-3-3ζ, NMR titration can be a quick way to test the binding/determine the binding site of small molecule or peptide 14-3-3 inhibitors [49,50]. Similarly, this may also be done for a number of recently discovered 14-3-3 protein-protein small-molecule stabilizers [49,50], which offer an alternative approach to modulating 14-3-3 activity.

## Materials and Methods

### Expression and purification of 14-3-3ζ

The human14-3-3ζ ORF purchased from GeneCopoeia was transferred into Gateway pDEST17 vector (Invitrogen). The 14-3-3ζΔC12, where the 12 C-terminal residues were deleted, was then generated from the full-length construct using QuikChange II Site-Directed Mutagenesis Kit (Strategene). Both full-length 14-3-3ζ and 14-3-3ζΔC12 were expressed as N-terminally His-tagged protein in *Escherichia coli* (*E*. *coli*) BL21 (DE3) pLysS in M9 medium. For unlabeled protein, protein expression was induced at an O.D._600_ of 0.9 by adding 0.75 mM IPTG at 18°C for 24 h. The ^15^N, ^13^C, ^2^H labeled 14-3-3ζΔC12 was produced by growing *E*. *coli* in deuterated M9 medium. Cells adapted to 70% D_2_O were used to inoculate a 1 L culture of M9 prepared in 100% D_2_O. Cells were grown at 37°C and overexpression was induced at an O.D._600_ of 0.6 with 0.75 mM IPTG. After a 60-h induction at 18°C, the cells were harvested. All 14-3-3ζ constructs used in this study were purified from the crude lysate by affinity chromatography using Ni Sepharose Fast Flow beads (Amersham Biosciences). The tag was then cleaved by overnight incubation with His-tagged tobacco etch virus (TEV) protease at 25°C. The cleaved 14-3-3ζ, which retains an N-terminal glycine, was purified by passing the mixture through Ni Sepharose Fast Flow beads. The final yields for the 14-3-3ζ proteins were ~50 mg per L of M9 media (including deuterated media).

### Peptide Synthesis

The Cby peptides used in this study were purchased from GenScript USA Inc. and the TUFTS University Core Facility. From GenScript USA Inc.: Cby 13-mer (NH_2_-^12^KTPPRKSASLSNL^24^-COOH), Cby S20D 13-mer (NH_2_-^12^KTPPRKSADLSNL^24^-COOH), Cby S20E 13-mer (NH_2_-^12^KTPPRKSAELSNL^24^-COOH), pCby 13-mer (NH_2_-^12^KTPPRKSApSLSNL^24^-COOH) and pCby 18-mer ((NH_2_-^12^KTPPRKSApSLSNLHSLDR^29^-COOH). From TUFTS: pCby 7-mer (Ac-^16^RKSApSLS^22^-NH_2_), pCby 11-mer (NH_2_-^12^KTPPRKSApSLS^22^-NH_2_), pCby S22P 7-mer (Ac-^16^RKSApSLP^22^-NH_2_), pCby S22P 13-mer (NH_2_-^12^KTPPRKSApSLPNL^24^-COOH) and pCby S22P 18-mer (NH_2_-^12^KTPPRKSApSLSNLHSLDR^29^-COOH). Peptides were dissolved and dialyzed in 50 mM sodium phosphate, 100 mM NaCl at pH 6.8 for ITC and NMR experiments.

### Crystallization and Structure Determination

Crystals grew within 2 months at 4°C in sitting drops containing 1 μL of complexed 14-3-3ζ (10 mg/mL) and phosphorylated Cby 18-mer peptide (protein:peptide ratio 1:4) in 20 mM Tris, pH 7.5, and 100 mM NaCl and 1 μL of reservoir solution containing 200 mM NH_4_HCO_2_ and 16–20% PEG 3350. The mother liquor with 15% glycerol served as the cryoprotectant for flash-cooling in liquid nitrogen. Data were collected at the Macromolecular Crystallographic Facility (University of Western Ontario). The structure was solved via rigid body molecular replacement, using Protein Data Bank entry 1QJA [37] as the starting model. The Cby peptide was built manually using *Coot* [51] and refinement was carried out using PHENIX [52].

### NMR Experiments

All NMR experiments were performed using ^15^N, ^13^C, ^2^H-labeled protein samples in 50 mM sodium phosphate, 100 mM NaCl at pH 6.8. Samples contained 10% D_2_O and 1 mM 2,2-dimethyl-2-sila-pentane-5-sulfonic acid (DSS) as ^1^H chemical shift reference. NMR experiments for the backbone resonance assignment of 14-3-3ζΔC12 were conducted at 25°C on a Bruker Avance 800 MHz (Singapore) spectrometer equipped with cryogenic probe. Sequential assignments were obtained from ^1^H-^15^N TROSY HSQC, HNCACB, HN(CO)CACB and ^15^N-NOESY-HSQC spectra. Data were processed using NMRPipe [53] and analyzed using CARA [54].

For the NMR titrations, either phosphorylated Cby 7-mer or 18-mer peptides were titrated into 600 μL of ~ 200 μM ^15^N, ^13^C, ^2^H labeled 14-3-3ζΔC12 until a 3:1 (peptide: 14-3-3ζΔC12) molar ratio was reached. A ^1^H-^15^N HSQC spectrum was collected for each titration point for a total of 12 points for the pCby WT 7-mer and 18-mer and 6 points for the pCby S22P 18-mer. All spectra were analyzed using NMRView [55].

### Isothermal titration calorimetry (ITC) experiments

ITC experiments were performed on a VP-ITC instrument (MicroCal) at 25°C. The protein and peptide samples were dialyzed into a buffer containing 50 mM sodium phosphate, 100 mM NaCl, 1 mM DTT at pH 6.8 and degassed before the experiments. In a typical experiment, 5 μL aliquots of ~1–2 mM peptide were titrated stepwise into the 1.4 mL sample cell containing ~100–200 μM 14-3-3ζ. The association constant (K_a_), molar binding stoichiometry (n) and the binding enthalpy (ΔH), entropy (ΔS) and Gibbs free energy (ΔG) were determined by fitting the binding isotherm to a one-site model with MicroCal Origin7 software. All ITC experiments were performed in duplicate. A duplicate set of thermodynamic derived binding parameters are found in S1 Table. Peptide and protein concentrations were determined from amino acid analysis (Amino Acid Analysis Facility, SickKids, Toronto, ON).

### Accession Numbers

The atomic coordinates for the 14-3-3ζ/Cby complex have been deposited in the Protein Data Bank under accession number 4WRQ. The ^1^H, ^15^N and ^13^Cα/β chemical shifts of the backbone resonances have been deposited in the BioMagResBank (http://www.bmrb.wisc.edu), under BMRB accession number 25231.

## Supporting Information

S1 Fig ITC thermograms for titrations of unphosphorylated or phospho-mimetic Cby peptides to 14-3-3ζ. (A) Cby WT 13-mer. (B) Cby S20D 13-mer. (C) Cby S20E 13-mer.
(TIF)

S2 Fig ITC thermograms for various phosphorylated WT and mutant Cby peptides titrated into 14-3-3ζ. (A) Cby 7-mer. (B) Cby 11-mer. (C) Cby 13-mer. (D) Cby 18-mer. (E) Cby S22P 7-mer (F) Cby S22P 13-mer. (G) Cby S22P 18-mer. (H) Cby L24A 18-mer.
(TIF)

S3 Fig A comparison of the orientation of 14-3-3ζ’s K49 side-chain in the 14-3-3ζ/Cby and 14-3-3ζ/Raf1 (PDB: 3CU8) complexes. Cby is represented by the green sticks and Raf1 by the light pink sticks. K49 in the 14-3-3ζ/Cby and 14-3-3ζ/Raf1 complexes is shown in yellow and dark pink, respectively.
(TIF)

S4 Fig ITC thermograms for the (A) WT Cby 18-mer and (B) Cby S22P 18-mer titrated to 14-3-3ζ K49A. (TIF)

S5 Fig ^1^H-^15^N TROSY-HSQC of ^2^H/^13^C/^15^N 14-3-3ζ in the absence (blue) and presence of a 1:1 molar ratio of the Cby 18-mer (red). Circled resonances represent the intense signals arising from 14-3-3ζ’s disordered C-terminal tail. A zoomed-in view displays the intense signals derived from 14-3-3ζ’s disordered C-terminal tail.
(TIF)

S6 Fig ITC thermograms for the (A) Cby 13-mer and (B) Cby 18-mer titrated to 14-3-3ζΔC12. (TIF)

S7 Fig Composite ^1^H_N_ and ^15^N chemical shift perturbation [Δω = ((Δδ^1^H)^2^ +(0.2*Δδ^15^N)^2^)^1/2^] analysis of 14-3-3ζΔC12 in the presence of three molar equivalents of the (A) Cby 7-mer, (A + B) Cby 18-mer and (B) Cby S22P 18-mer. Due to the crowding of some peaks, the chemical shifts of some residues could not be confidently traced and were excluded from the analysis.
(TIF)

S8 Fig Observed chemical shifts for 14-3-3ζΔC12 residues 204–215. Resonances shown for each residue include the apo state (black), Cby 18-mer bound-state (blue) and the pCby 7-mer bound-state (red), at a 3:1 (pCby: 14-3-3ζΔC12) ratio. *Residue Y211 is displayed at a 1.25:1 (Cby: 14-3-3ζΔC12) ratio as it broadens out to disappearance at a 3:1 ratio with the Cby 7-mer. The Cby-18-mer bound-state is shown in cyan with the 7-mer bound-state in magenta.
(TIF)

S1 Table Duplicate set of thermodynamic parameters for the binding of phosphorylated Cby peptides to 14-3-3ζ. (DOCX)
