# Supplementary figures and images for: Structural Analysis of the 14-3-3ζ/Chibby Interaction Involved in Wnt/β-Catenin Signaling
Source: PLoS One. 2015 Apr 24;10(4):e0123934. doi: 10.1371/journal.pone.0123934 (PMC4409382; doi:10.1371/journal.pone.0123934)

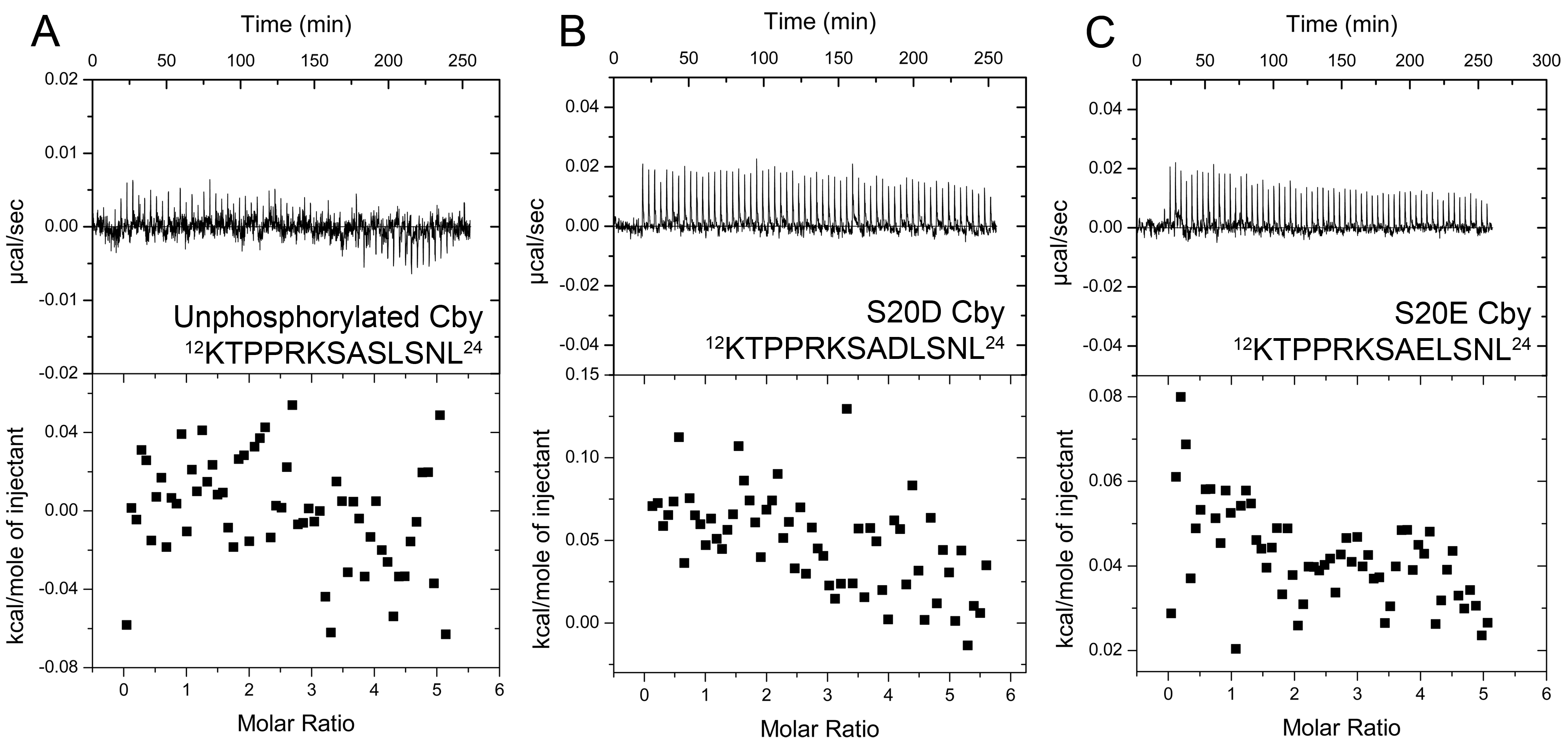

Supplement: S1 Fig — (A) Cby WT 13-mer. (B) Cby S20D 13-mer. (C) Cby S20E 13-mer. (TIF) [file pone.0123934.s001.tif]

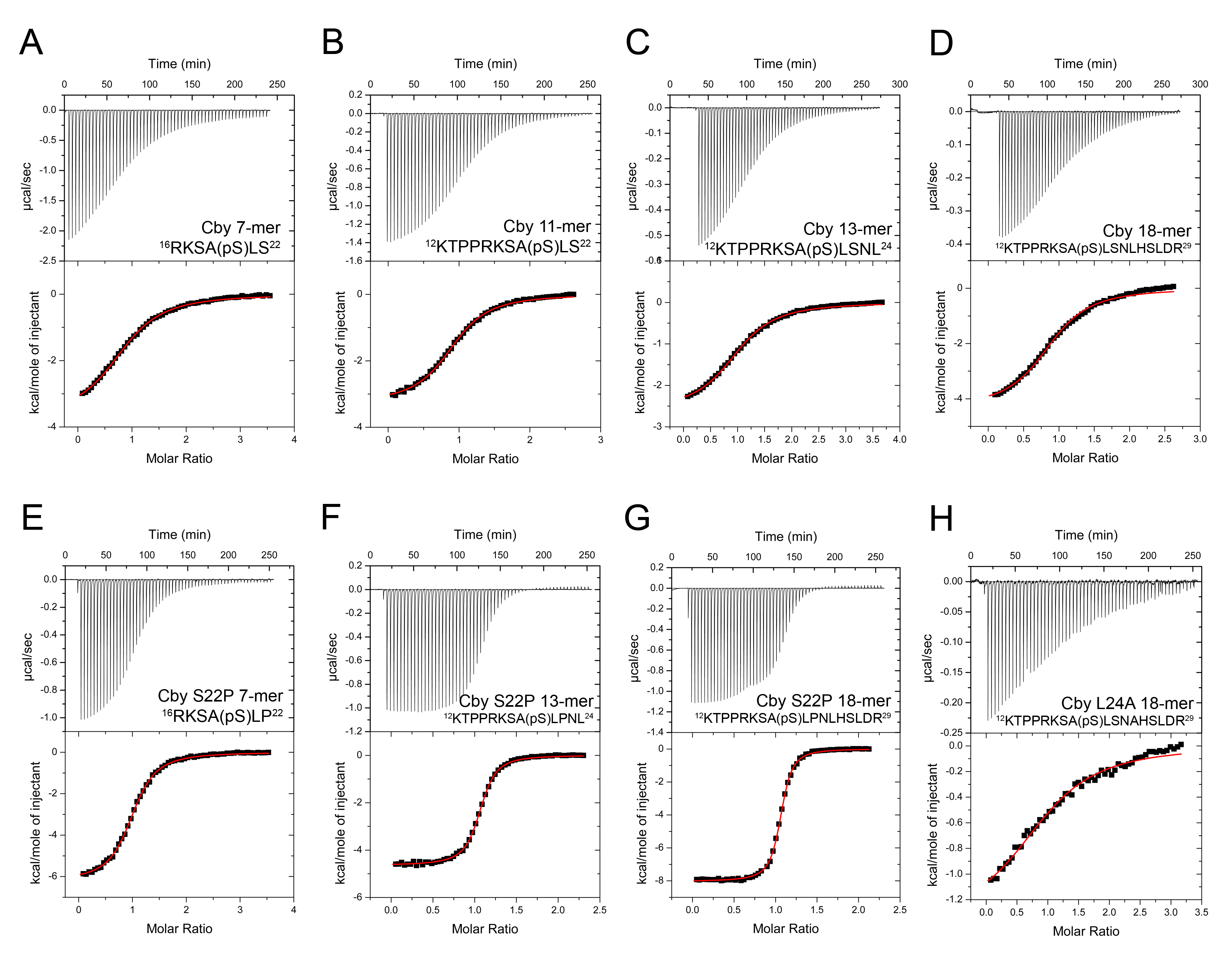

Supplement: S2 Fig — (A) Cby 7-mer. (B) Cby 11-mer. (C) Cby 13-mer. (D) Cby 18-mer. (E) Cby S22P 7-mer (F) Cby S22P 13-mer. (G) Cby S22P 18-mer. (H) Cby L24A 18-mer. (TIF) [file pone.0123934.s002.tif]

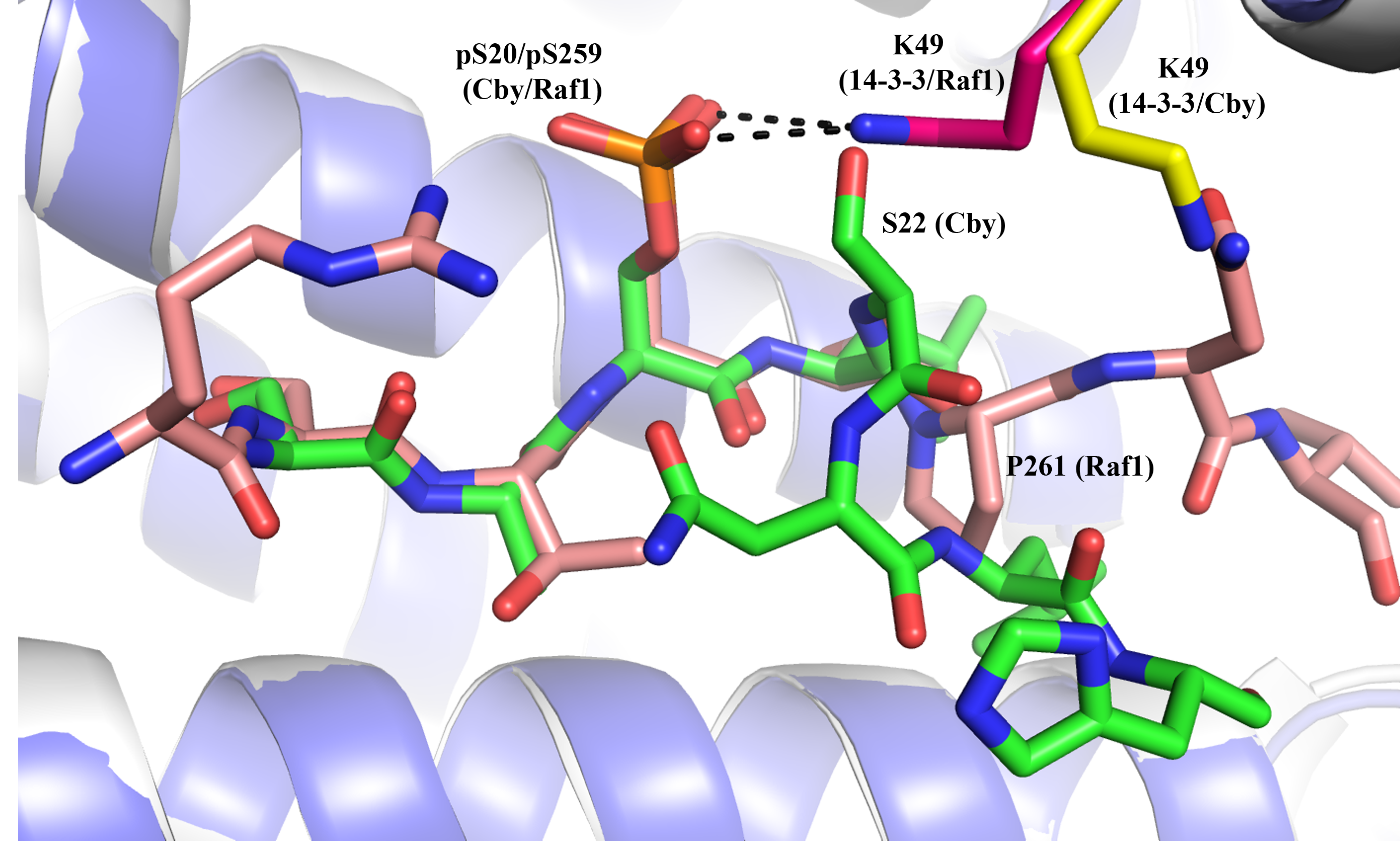

Supplement: S3 Fig — Cby is represented by the green sticks and Raf1 by the light pink sticks. K49 in the 14-3-3ζ/Cby and 14-3-3ζ/Raf1 complexes is shown in yellow and dark pink, respectively. (TIF) [file pone.0123934.s003.tif]

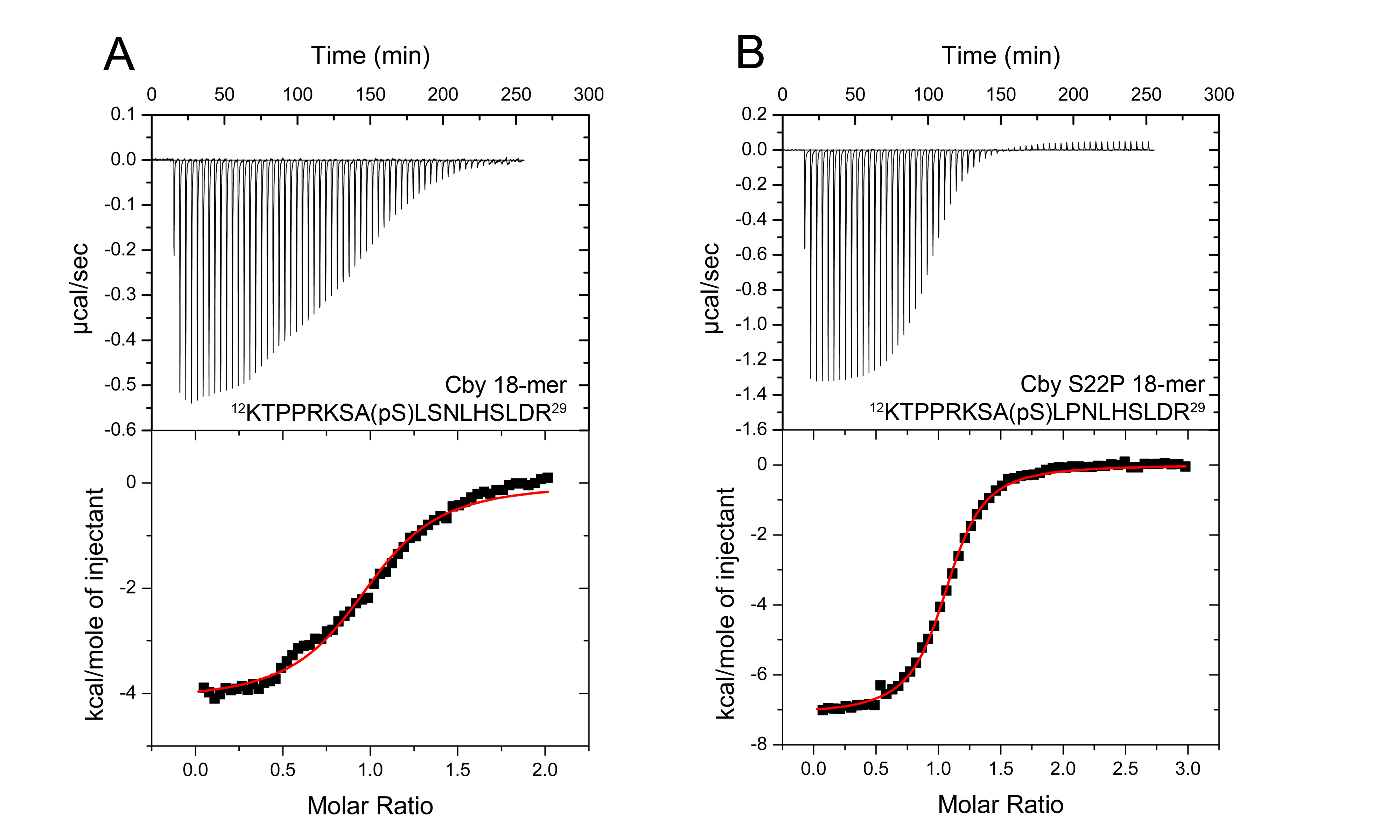

Supplement: S4 Fig — (TIF) [file pone.0123934.s004.tif]

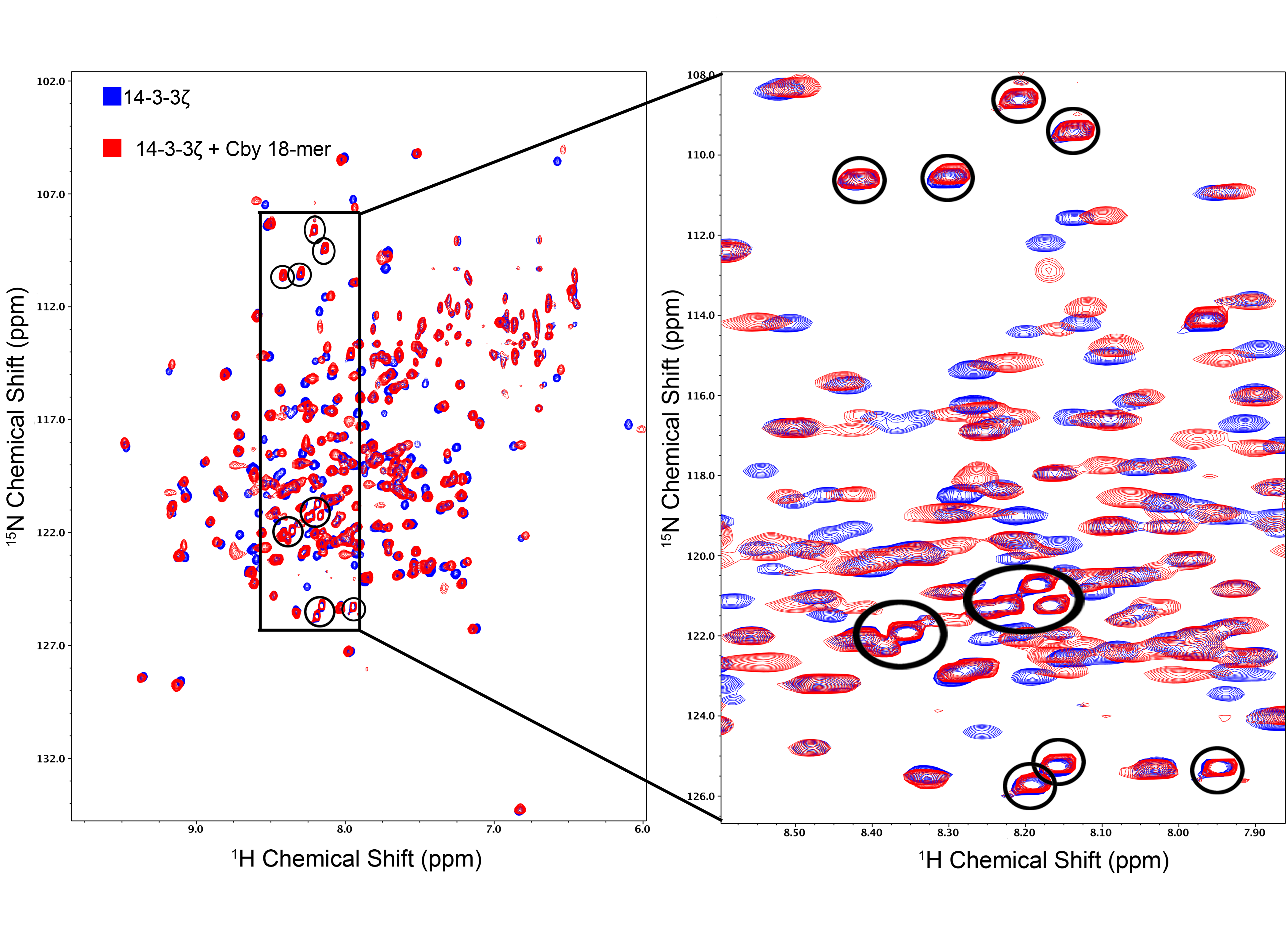

Supplement: S5 Fig — Circled resonances represent the intense signals arising from 14-3-3ζ’s disordered C-terminal tail. A zoomed-in view displays the intense signals derived from 14-3-3ζ’s disordered C-terminal tail. (TIF) [file pone.0123934.s005.tif]

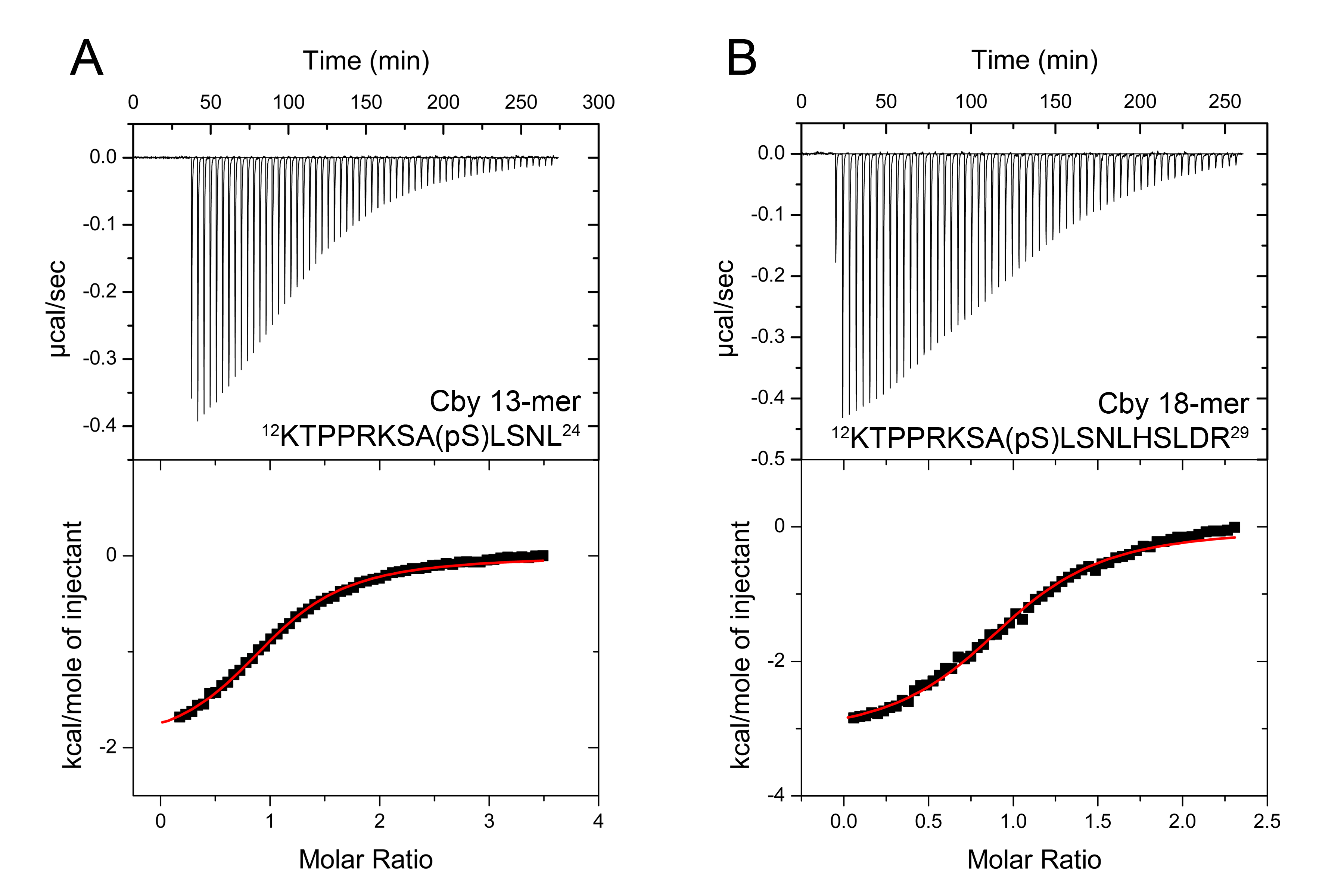

Supplement: S6 Fig — (TIF) [file pone.0123934.s006.tif]

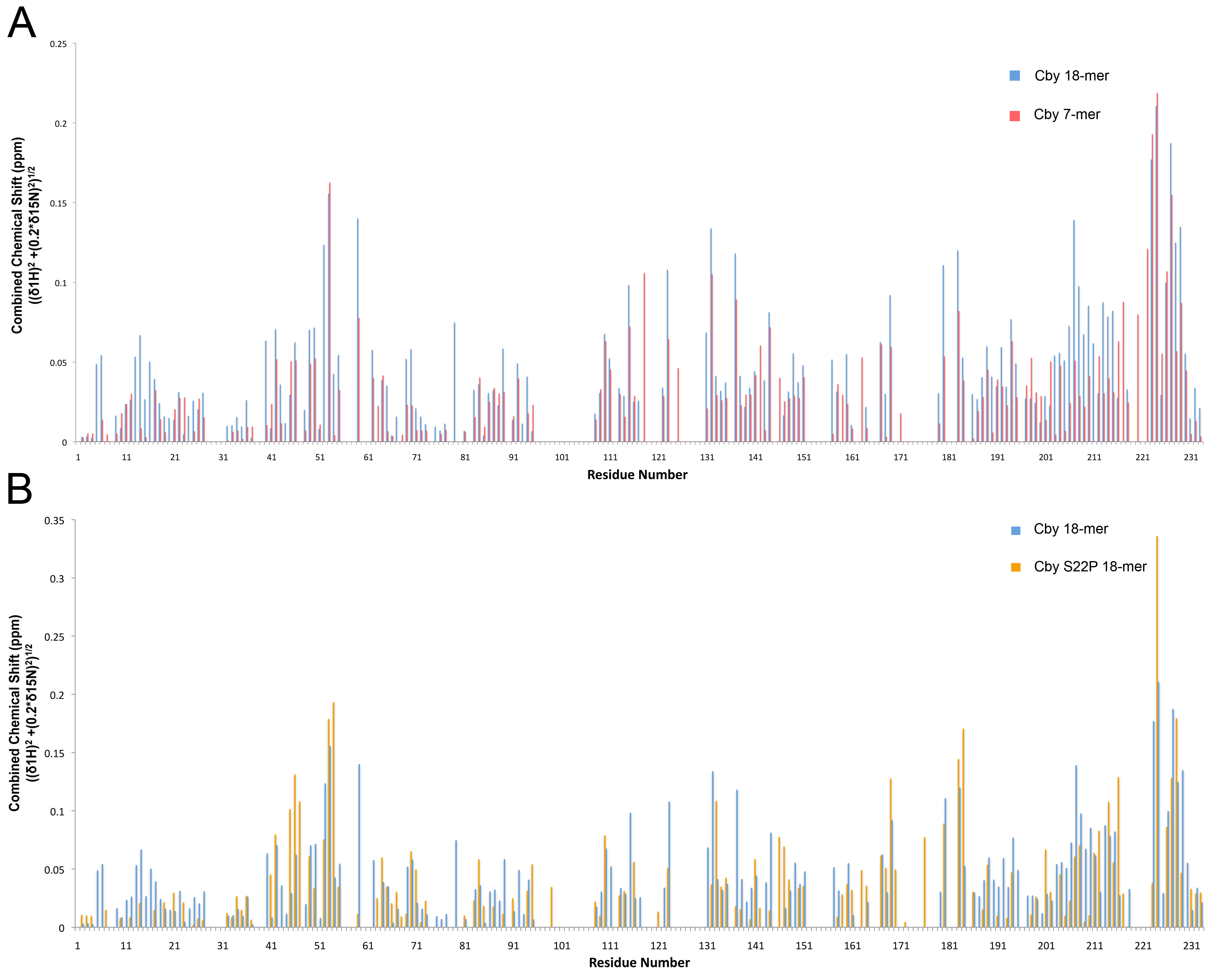

Supplement: S7 Fig — Due to the crowding of some peaks, the chemical shifts of some residues could not be confidently traced and were excluded from the analysis. (TIF) [file pone.0123934.s007.tif]

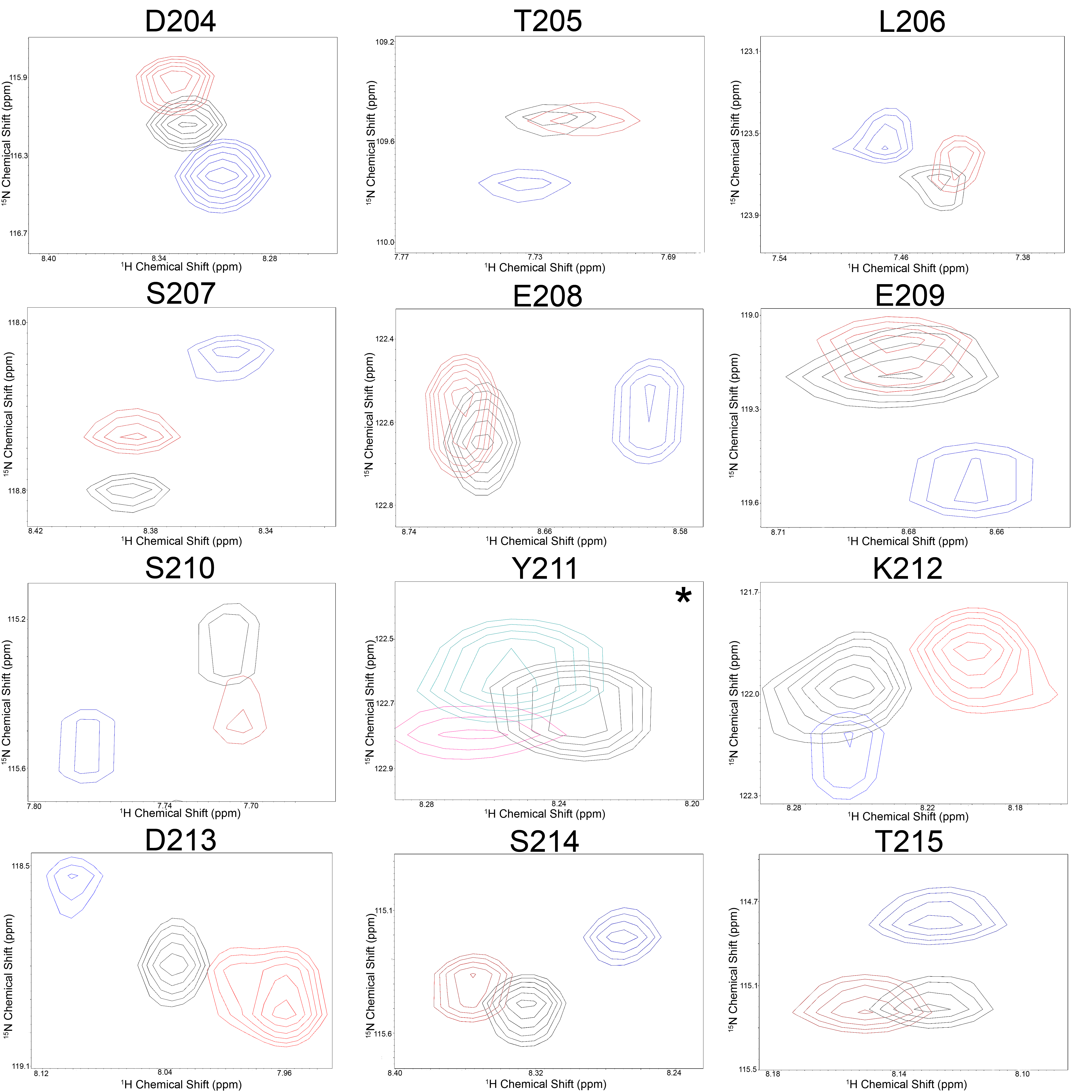

Supplement: S8 Fig — Resonances shown for each residue include the apo state (black), Cby 18-mer bound-state (blue) and the pCby 7-mer bound-state (red), at a 3:1 (pCby: 14-3-3ζΔC12) ratio. *Residue Y211 is displayed at a 1.25:1 (Cby: 14-3-3ζΔC12) ratio as it broadens out to disappearance at a 3:1 ratio with the Cby 7-mer. The Cby-18-mer bound-state is shown in cyan with the 7-mer bound-state in magenta. (TIF) [file pone.0123934.s008.tif]
