# Supplementary material for: Structural Analysis of the 14-3-3ζ/Chibby Interaction Involved in Wnt/β-Catenin Signaling
Source: PLoS One. 2015 Apr 24;10(4):e0123934. doi: 10.1371/journal.pone.0123934 (PMC4409382; doi:10.1371/journal.pone.0123934)
Supplement: S1 Table — (DOCX) [file pone.0123934.s009.docx]

| Peptide | n^a^ | K_d_^b^  (10^-6^ M) | ∆H^b^  (kcal/mol) | T∆S^b^ (kcal/mol) | ∆G^b^  (kcal/mol) |
| --- | --- | --- | --- | --- | --- |
| ***In ITC Cell: 14-3-3* ζ** |  |  |  |  |  |
| *WT Cby* |  |  |  |  |  |
| Cby 7-mer  ^16^RKSA(pS)LS^22^ | 0.94 | 47.6 ± 1.6 | -3.70 ± 0.04 | 2.20 | -5.90 ± 0.02 |
| Cby 11-mer  ^12^KTPPRKSA(pS)LS^22^ | 0.96 | 17.5 ± 0.6 | -3.27 ± 0.02 | 3.22 | -6.49 ± 0.02 |
| Cby 13-mer  ^12^KTPPRKSA(pS)LSNL^24^ | 1.07 | 12.8 ± 0.5 | -2.60 ± 0.03 | 4.07 | -6.67 ± 0.02 |
| Cby 18-mer  ^12^KTPPRKSA(pS)LSNLHSLDR^29^ | 0.94 | 4.9 ± 0.3 | -4.33 ± 0.06 | 2.91 | -7.24 ± 0.04 |
|  |  |  |  |  |  |
| *Cby Mutants* |  |  |  |  |  |
| Cby 7-mer S22P  ^16^RKSA(pS)LP^22^ | 1.02 | 3.2 ± 0.1 | -6.21 ± 0.02 | 1.29 | -7.50 ± 0.02 |
| Cby 13-mer S22P  ^12^KTPPRKSA(pS)LPNL^24^ | 1.02 | 1.3 ± 0.04 | -4.70 ± 0.01 | 3.33 | -8.03 ± 0.02 |
| Cby 18-mer S22P  ^12^KTPPRKSA(pS)LPHSLDR^29^ | 1.08 | 0.38 ± 0.01 | -7.97 ± 0.01 | 0.79 | -8.76 ± 0.02 |
| Cby 18-mer L24A  ^12^KTPPRKSA(pS)LSNAHSLDR^29^ | 1.05 | 25.0 ± 2.5 | -1.37 ± 0.05 | 4.91 | -6.28 ± 0.06 |
|  |  |  |  |  |  |
| ***In ITC Cell: 14-3-3* ζ K49A** |  |  |  |  |  |
| Cby 18-mer WT  ^12^KTPPRKSA(pS)LSNLHSLDR^29^ | 1.07 | 5.5 ± 0.6 | -3.76 ± 0.07 | 3.41 | -7.17 ± 0.06 |
| Cby 18-mer S22P  ^12^KTPPRKSA(pS)LPHSLDR^29^ | 0.97 | 1.8 ± 0.1 | -6.67 ± 0.04 | 1.17 | -7.84 ± 0.03 |
|  |  |  |  |  |  |
| ***In ITC Cell: 14-3-3 ζ ΔC12*** |  |  |  |  |  |
| Cby 13-mer  ^12^KTPPRKSA(pS)LSNL^24^ | 1.06 | 14.5 ± 0.6 | -2.08 ± 0.03 | 4.52 | -6.60 ± 0.02 |
| Cby 18-mer  ^12^KTPPRKSA(pS)LSNLHSLDR^29^ | 1.02 | 8.5 ± 0.5 | -3.15 ± 0.04 | 3.77 | -6.92 ± 0.03 |

^a^ Binding stoichiometry of monomeric 14-3-3 and Cby peptide.

^b^K_d_ is the dissociation constant. ∆H, ∆S and ∆G are the change in enthalpy, entropy and Gibbs free energy upon binding at T=298.15 K, respectively.
